# Naringin regulates mitochondrial dynamics to protect against acetaminophen-induced hepatotoxicity by activating the AMPK/Nrf2 signaling pathway *in vitro*

**DOI:** 10.1590/1414-431X2022e12040

**Published:** 2022-10-17

**Authors:** Qiao Wu, Pengfei Yu, Yanzhen Bi, Zhijie Li, Wei Guo, Yu Chen, Zhongping Duan

**Affiliations:** 1 Infection Center, Beijing Tiantan Hospital, Capital Medical University, Beijing, China; 2 Fourth Department of Liver Disease (Difficult & Complicated Liver Diseases and Artificial Liver Center), Beijing You'an Hospital Affiliated to Capital Medical University, Beijing, China; 3 Beijing Municipal Key Laboratory of Liver Failure and Artificial Liver Treatment Research, Beijing, China; 4 Hepatobiliary Surgery Center, The Fifth Medical Center of PLA General Hospital, Beijing, China

**Keywords:** Naringin, Acetaminophen, Hepatotoxicity, Mitochondrial dynamics, Antioxidant enzyme, Nrf2

## Abstract

Naringin (Nar) has been reported to exert potential hepatoprotective effects against acetaminophen (APAP)-induced injury. Mitochondrial dysfunction plays an important role in APAP-induced liver injury. However, the protective mechanism of Nar against mitochondrial damage has not been elucidated. Therefore, the aim of this study was to investigate the hepatoprotective effects of Nar against APAP and the possible mechanisms of actions. Primary rat hepatocytes and HepG2 cells were utilized to establish an *in vitro* model of APAP-induced hepatotoxicity. The effect of APAP and Nar on cell viability was evaluated by a CCK8 assay and detection of the concentrations of alanine aminotransferase, aspartate aminotransferase, and lactate dehydrogenase. The cellular concentrations of biomarkers of oxidative stress were measured by ELISA. The mRNA expression levels of APAP-related phase II enzymes were determined by real-time PCR. The protein levels of Nrf2, phospho (p)-AMPK/AMPK, and biomarkers of mitochondrial dynamics were determined by western blot analysis. The mitochondrial membrane potential (MMP) was measured by high-content analysis and confocal microscopy. JC-1 staining was performed to evaluate mitochondrial depolarization. Nar pretreatment notably prevented the marked APAP-induced hepatocyte injury, increases in oxidative stress marker expression, reductions in the expression of phase II enzymes, significant loss of MMP, mitochondrial depolarization, and mitochondrial fission *in vitro*. In conclusion, Nar alleviated APAP-induced hepatocyte and mitochondrial injury by activating the AMPK/Nrf2 pathway to reduce oxidative stress *in vitro*. Applying Nar for the treatment of APAP-induced liver injury might be promising.

## Introduction

Acetaminophen (APAP) is widely used as an over-the-counter analgesic and antipyretic drug (1). APAP is responsible for 46% of all acute liver failure (ALF) toxicity in the United States (2,3) and 40-70% of all cases in the United Kingdom and Europe (1). Currently, oxidative stress and mitochondrial malfunction are considered the main cellular processes mediating APAP-induced hepatocytotoxicity (4-6). APAP metabolism involves hepatic phase I and II enzyme-related detoxification pathways (7). Approximately 85% of APAP is converted into APAP-gluc and APAP-sulfur via liver II binding enzymes (including UDP-glucuronosyltransferase (UGT) and sulfotransferase (SULT)) and is then excreted in the urine (8). It is widely believed that the toxic product of APAP, N-acetyl-p-benzoquinone imine (NAPQI), is produced mainly via the hepatic phase I metabolism through the cytochrome P-450 pathway (mainly through CYP2E1) (9). Under conditions of excessive APAP, phase II metabolism is saturated, but overproduction of NAPQI uses up liver glutathione (GSH) and modifies cellular proteins (9). Studies have shown that protein adducts in mitochondria disrupt the electron transport chain, generate oxidative stress and trigger changes in mitochondrial membrane permeability, causing pore opening, changes in the MMP, and mitochondrial fission (7). Eventually, mitochondrial malfunction leads to cell necrosis. Therefore, inhibiting oxidative stress and ameliorating mitochondrial malfunction may play a crucial role in reducing APAP-induced acute liver damage (10). N-acetylcysteine (NAC) is an acknowledged antioxidant that has a valid therapeutic effect on early APAP-induced acute liver damage. However, its use is limited by its narrow therapeutic window and side effects (11). Therefore, there is an obvious need for new treatments that can prevent APAP-induced acute liver damage.

Natural products, including herbs, have contributed significantly to drug discovery (11). Compared with chemical compound drugs, natural products have the advantages of few side effects, low toxicity after long-term use, and variable bioavailability and biological activity. Naringin (Nar) has been approved for clinical trials by the Chinese Food and Drug Administration over the past 20 years (12). Clinical studies (registration numbers: CTR20130704 for single-dose studies and CTR20190127 for multiple-dose studies, http://www.chinadrugtrials.org.cn/eap/main) were conducted. Recently, numerous studies have shown that natural products exert a protective effect against APAP-induced hepatotoxicity and play multiple roles in inflammation, the oxidant/antioxidant balance, and damage responses (13,14). The key mechanisms of APAP-induced liver injury include APAP metabolism, oxidative stress, and mitochondrial dynamics, which can be regulated by natural products. Nar is a dihydroflavonoid derived mainly from *Citrus grandis (L.) Osbeck* and the immature or nearly mature dry outer peel of grapefruit (*C. paradisi Macfad*). Nar is the main active component of ganshuang granule, an herbal prescription known for its hepatoprotective effects (15). Nar not only exhibits antioxidant activity (15-17) but also exerts inhibitory effects on cytochrome P450 (18). Moreover, Nar can ameliorate APAP-induced oxidative stress and liver tissue damage *in vivo* (19,20). Although both Nrf2 and AMPK proteins are reported to be activated by Nar, no reports have addressed the mechanism by which Nar affects the AMPK/Nrf2 signaling pathway (21,22). Since mitochondrial dysfunction plays an important role in APAP-induced liver injury, we investigated the hepatoprotective mechanism of Nar from the perspective of regulating mitochondrial dynamics via AMPK/Nrf2 signaling pathway activation *in vitro*.

## Material and Methods

### Ethical approval

The Capital Medical University Animal Experiments and Experimental Animals Management Committee (AEEI-2020-076) approved all protocols for feeding, anesthesia, blood and tissue sampling, and euthanasia of animals.

### Experimental animal care

Male Sprague-Dawley rats weighing 200-220 g were used. Experimental animals were fed ad libitum and housed in steel cages with an average temperature of 22±2°C and 12-h day and night cycles that were adjusted automatically.

### Reagents and antibodies

Dulbecco’s modified Eagle’s medium (DMEM), RPMI 1640 medium, fetal bovine serum, penicillin/streptomycin (10,000 units/mL penicillin and 10,000 µg/mL streptomycin), and 0.25% trypsin-EDTA were purchased from Gibco, Thermo Fisher Scientific, Inc. (USA). JC-1 dye was obtained from Invitrogen, Thermo Fisher Scientific, Inc. (USA). Nar, APAP, dorsomorphin (Dor), and brusatol (Bru) were obtained from MedChemExpress (USA) and dissolved in dimethyl sulfoxide (DMSO). A malondialdehyde (MDA) assay kit (TBA method), superoxide dismutase (SOD) assay kit (WST-1 method), reduced GSH assay kit, and glutamate dehydrogenase (GDH) test kit were obtained from Nanjing Jiancheng Bioengineering Institute (China). A reactive oxygen species (ROS) assay kit was obtained from Beyotime (China). A CCK8 Cell Proliferation and Cytotoxicity Assay Kit was obtained from Solarbio (China).

Antibodies specific for β-actin were purchased from Proteintech Group, Inc. (China). Antibodies specific for mitofusin protein 1 (Mfn1), optic atrophy type 1 (Opa1), phospho (p)-dynamin-related protein 1 (Drp1) (Ser616), total Drp1, AMPK, p-AMPK (Thr172), Nrf2, and GAPDH were purchased from Cell Signaling Technology (USA).

### Isolation and culture of primary rat hepatocytes

After anesthetization with pentobarbital sodium, the liver portal vein was exposed and a 22G type Y catheter was inserted (BD, USA) to provide a perfusion inlet to the vasculature of the liver. HBSS was pumped in at a rate of 18 mL/min to flush the rat liver. After 15 min, type IV collagenase was pumped in at the same rate. The liver was cut into pieces, and hepatocytes were isolated. Hepatocyte medium (ScienCell, USA) was added to the hepatocyte precipitate and mixed gently. The sample was centrifuged twice at 50 *g* for two minutes each at 4°C. After seeding, cells (4×10^5^ cells/well) were cultured in a 6-well plate treated with tail collagen in a cell incubator at 37°C in 5% CO_2_. The solution was changed once at 12 h, and the morphology of hepatocytes was observed prior to treatment.

### Culture of HepG2 cells

HepG2 cells were maintained in our laboratory and cultured in DMEM supplemented with 10% fetal bovine serum at 37°C in a cell incubator with 95% humidity and 5% CO_2_ (Thermo Forma, USA). EDTA-trypsin was used to digest cells when adherent to the wall at 85∼90% confluence, and the cell survival rate was greater than 90% for 1:4 subculture.

### Establishment of *in vitro* cell models

The original generation of primary rat hepatocytes or HepG2 cells was used in the follow-up experiments. In all experiments, cells were seeded at a proper density as needed and used at a confluence of 70-80%. Cells were randomly assigned to five groups: 1) the control group, which was cultured in normal culture medium; 2) the injury group (APAP group), which was treated with 20 mM APAP for 24 h; and 3) the three Nar groups, which were pretreated with 1, 10, or 100 μM Nar for 8 h before APAP treatment. In addition, HepG2 cells were treated with dorsomorphin (30 μM), which inhibits AMPK phosphorylation, or brusatol (200 nM), an Nrf2 inhibitor, according to the experimental design.

### CCK8 assay

For cell viability assays, primary rat hepatocytes or HepG2 cells were plated onto a 96-well plate at 1×10^4^ cells/mL in 100 μL of medium per well and incubated for 24 h. All cells were attached to the wall. After the plate was prepared as described in establishment of *in vitro* cell models, CCK8 working solution was added, and the absorbance at a wavelength of 450 nm was then measured in an ELISA plate reader (Bio-Rad, USA).

### ALT, AST, LDH, and GDH assays

The supernatants of the control group and the APAP group were collected. The relevant experimental steps were performed according to the assay methods and instructions. The levels of alanine aminotransferase (ALT), aspartate aminotransferase (AST), lactate dehydrogenase (LDH), and glutamate dehydrogenase (GDH) were measured in a multifunctional plate reader.

### Analysis of GSH, SOD, MDA, and ROS

The precipitated cells were washed 1-2 times with PBS and lysed with RIPA buffer. Then, the supernatant was taken, and the relevant experimental steps were performed according to the test methods and instructions. The concentrations of MDA, GSH, and ROS and the activity of SOD in cells were measured in a multifunctional plate reader.

### Measurement of the MMP

TMRE and MitoTracker Green (Invitrogen) were added to the different groups of cells, and the cells were incubated in a cell culture chamber for 30 min at 37°C. After 3 washes with HBSS, normal medium was added, and images were acquired by confocal microscopy.

After 8 h of Nar pretreatment and 1-2 washes with HBSS, MMP dye (Image-iT-TM TMRM reagent, 1:1000 dilution; Invitrogen) was added to the cells, and the cells were incubated in the cell culture chamber for 0.5 h at 37°C. The cells were washed 3 times with HBSS, normal medium or 20 mM APAP was added, and images of high-content analysis were acquired within 3 h.

Mitochondrial depolarization was detected using a mitochondria-specific cationic dye, JC-1, which is a new type of cationic carbocyanine dye that aggregates in mitochondria. HepG2 cells were pretreated with 1, 10, or 100 μM Nar for 8 h before APAP treatment. After 8 h of incubation with APAP, 10 μg/mL JC-1 was added and incubated in a 37°C incubator for 10 min. Then, the charging solution was replaced with fresh medium. We used fluorescence microscopy to detect and fluorescence microplate reader to analyze the fluorescence signal. A reduction in the red/green fluorescence intensity ratio indicated mitochondrial depolarization.

### Western blot analysis

All samples were collected, and total protein was extracted for concentration determination by the BCA method. Proteins (34 µg of protein per sample) were separated by 30% acrylamide-methylene bisacrylamide gel electrophoresis. Membrane transfer was performed with the wet transfer method. After transfer, Lichun red staining reagent was used to stain the membrane, and the transfer efficiency was visualized. The membrane was incubated with the monoclonal antibody diluted with 5% BSA-TBST overnight at 4°C. The next day, the membrane was washed and incubated with the secondary antibody with shaking for 40 min at room temperature and washed 3 times with TBST. A chemiluminescence solution was used for chemical color development and imaging.

### Real-time fluorescent quantitative polymerase chain reaction (RT-qPCR)

All groups of cells were collected, and total RNA was extracted using TRIzol reagent. cDNA was synthesized from 1 g RNA with primers and an AMV reverse transcriptase system. An ABI StepOnePlus real-time PCR system (TaKaRa, Japan) was used for real-time reverse transcription PCR with SYBR Green. The primers provided by Shanghai Sangon Company were used and are listed in Table 1. The relative expression levels of target genes were calculated by using the ΔΔCt method with the housekeeping gene GAPDH as the internal control.

**Table 1:** Primers used in this study.

Gene	Forward	Reverse
hGAPDH	GGACTCATGACCACAGTCCA	TCAGCTCAGGGATGACCTTG
hUGT1A1	CGCCTCTCCAGCCTTCACAAG	GTCAGCACGACGGCCAAGAG
hUGT1A3	GCCAACAGGAAGCCACTATC	CAGCAATTGCCATAGCTTTC
hUGT1A6	CCAGTGCCGTATGACCAAGAAGAG	GGAGGCTCTGGCAGTTGATGAAG
hUGT2B7	GCAGCAGAATACAGCCATTGGATG	GCTGAAGATGCCAGTACAGTCACC
hGSTα1	TCCAGCTTCCCTCTGCTGAA	GGCTGCCAGGCTGTAGAAAC
hGSTM1	GCCTGCTCCTGGAATACACAGAC	GCAATGTAGCACAAGATGGCGTTG
hSULT1A1	TGGTTCAGCACACGTCGTTCAAG	CATCTTCTCCGCATAGTCCGCATC
hSULT2A1	GAGATTCTCTGCCTGATGCACTCC	ATCACCTTGGCCTTGGAACTGAAG
hCYP2E1	GCCGACATCCTCTTCCGCAAG	CTGTGGCTTCCAGGCAAGTAGTG
rGAPDH	GACATGCCGCCTGGAGAA	AGCCCAGGATGCCCTTTAGT
rCYP2E1	CGCTTCGGGCCAGTGTTCAC	GTAGCACCTCCTTGACAGCCTTG

Genes: The prefix “h” indicates the human primer, and the prefix “r” indicates the rat primer.

### Data analysis

All results are reported as means±SD values. SPSS 23.0 software (IBM, USA) was used for statistical analysis. Differences among groups were compared by one-way analysis of variance (ANOVA), and pairwise comparisons were performed with *post hoc* Bonferroni tests. A P-value of <0.05 was defined as statistically significant.

## Results

### Nar ameliorated APAP-induced hepatotoxicity and increased the viability of primary rat hepatocytes and HepG2 cells

**Figure 1:**
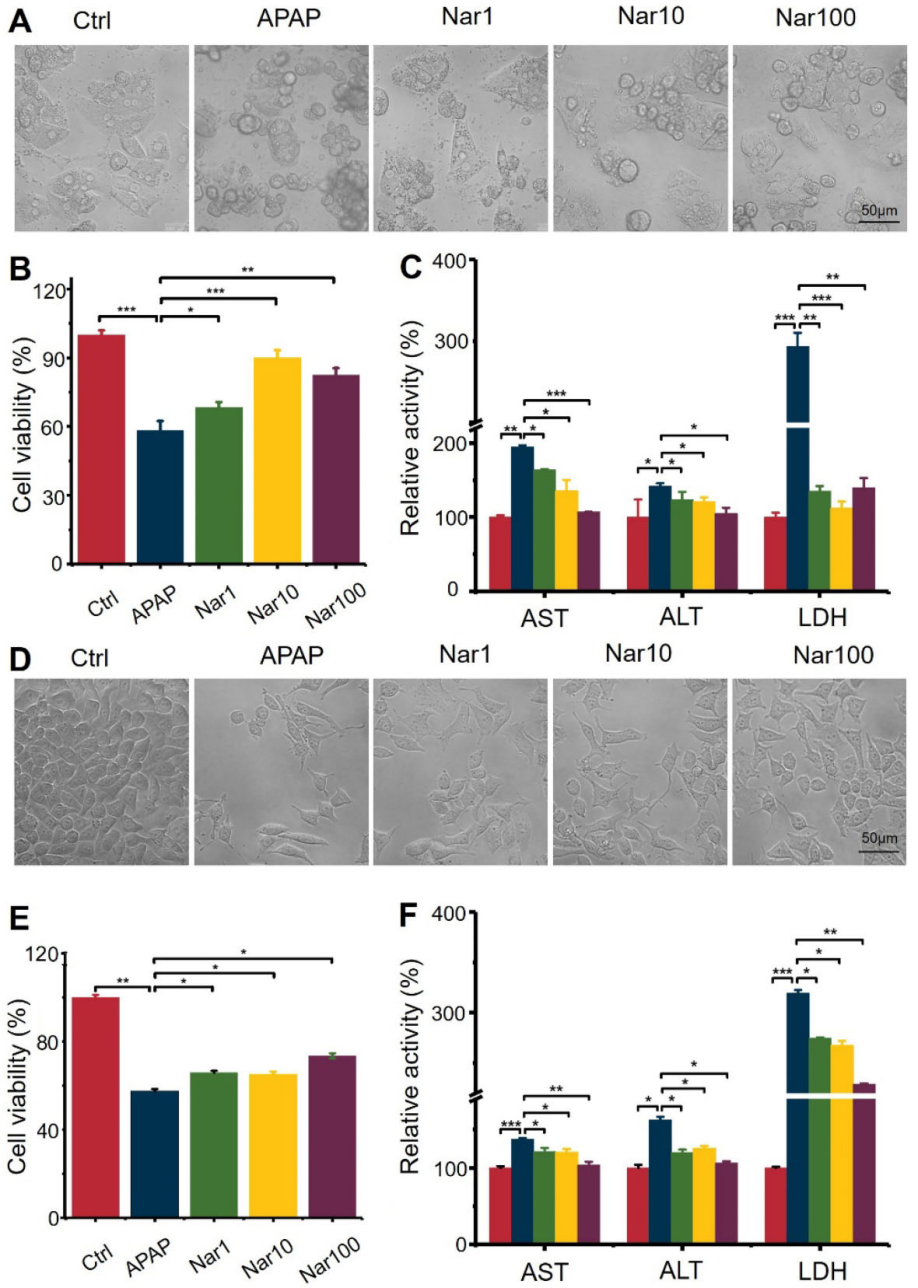
Cells pretreated with different concentrations of naringin (Nar) (1, 10, or 100 µM) for 8 h were exposed to acetaminophen (APAP; 20 mM) for 24 h. **A** and **D**, Cell morphology of the different groups, as assessed by microscopy (scale bar 50 μm). **B** and **E**, A CCK8 assay was used to determine the survival rate of primary rat hepatocytes and HepG2 cells. **C** and **F**, Aspartate aminotransferase (AST), alanine aminotransferase (ALT), and lactate dehydrogenase (LDH) levels were measured by ELISA to assess cell damage. Data are reported as means±SD (n=3). *P<0.05, **P<0.01, and ***P<0.001 *vs* the APAP group (ANOVA).

As shown in Figure 1A and D, cells in the control group showed good growth, clear background, firm cell apposition, were flattened, with high transparency and uniform shading. Cells in the model group (APAP, 20 mmol/L) showed significant apoptosis, reduced cell numbers, and reduced rounded cytosomes. Compared with the APAP group, the cells in the Nar group showed a significantly improved state, with a significant recovery of the ability to adhere to the wall, an enlarged cytosol, and most cells were shuttle-shaped. The morphological changes of primary hepatocytes and HepG2 cells showed that Nar significantly reduced the damaging effect of APAP and improved cell survival to a certain extent, indicating that Nar had a certain protective effect on liver cells damaged by APAP. The ALT, AST, and LDH activity levels in the culture supernatant were also significantly increased and cell viability was significantly decreased by administration of APAP compared with control cells (Figure 1B, C, E, and F). In contrast, the lower ALT, AST and LDH activities reduced the hepatotoxic effects of APAP in cells pretreated with Nar.

### Nar suppressed APAP-induced oxidative stress in primary rat hepatocytes and HepG2 cells

**Figure 2:**
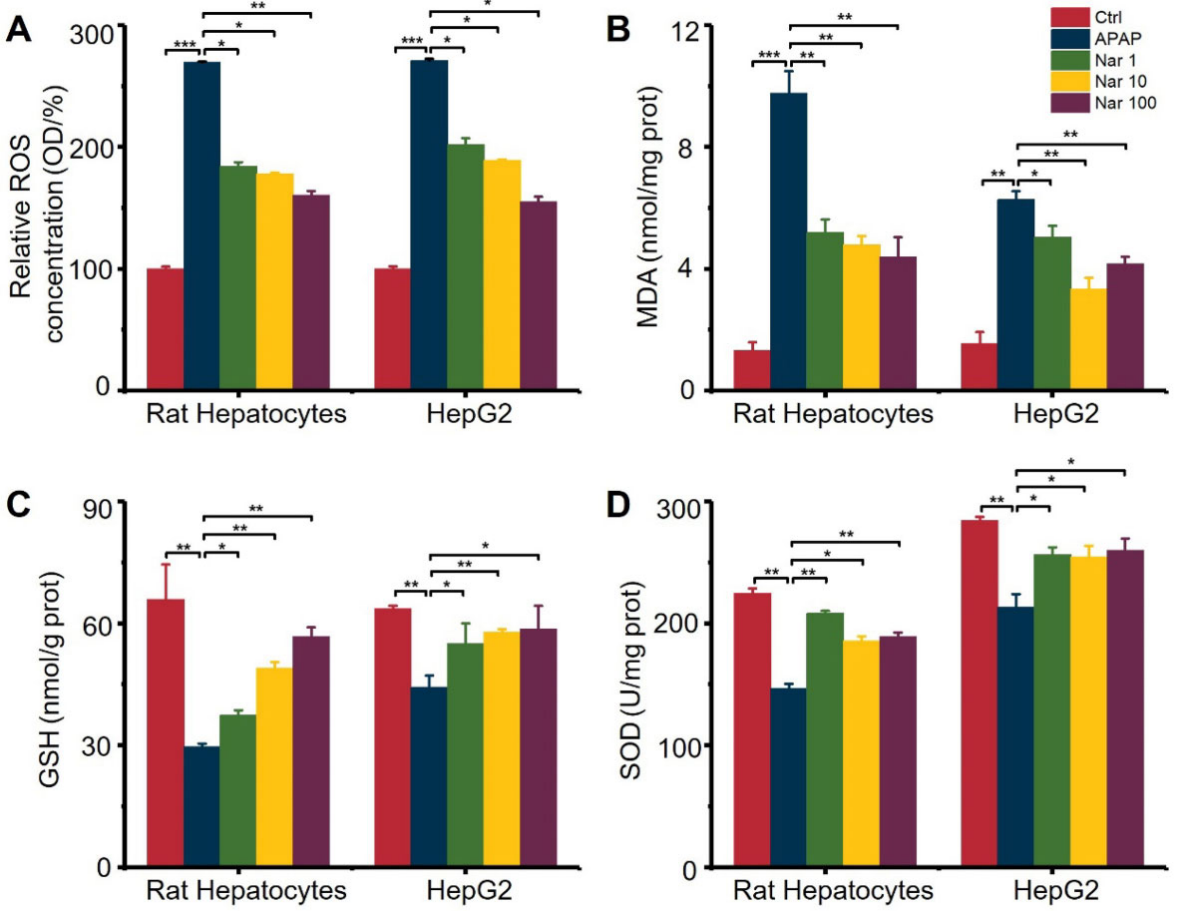
Effect of naringin (Nar) on oxidative stress induced by acetaminophen (APAP) in primary rat hepatocytes and HepG2 cells. The concentration of (**A**) reactive oxygen species (ROS), (**B**) malondialdehyde (MDA), (**C**) glutathione (GSH), and (**D**) superoxide dismutase (SOD) were detected by ELISA. Data are reported as means±SD (n=3). *P<0.05, **P<0.01, and ***P<0.001 (ANOVA).

Significantly increased concentrations of ROS and MDA and reduced concentrations of GSH resulted from the addition of APAP, indicating that APAP induced oxidative stress in hepatocytes. Moreover, APAP decreased the SOD activity level by 35 and 25%, respectively. In the Nar pretreatment groups, APAP-induced oxidative stress was significantly reduced (Figure 2).

### Nar suppressed the expression of CYP2E1 in primary rat hepatocytes and HepG2 cells

**Figure 3:**
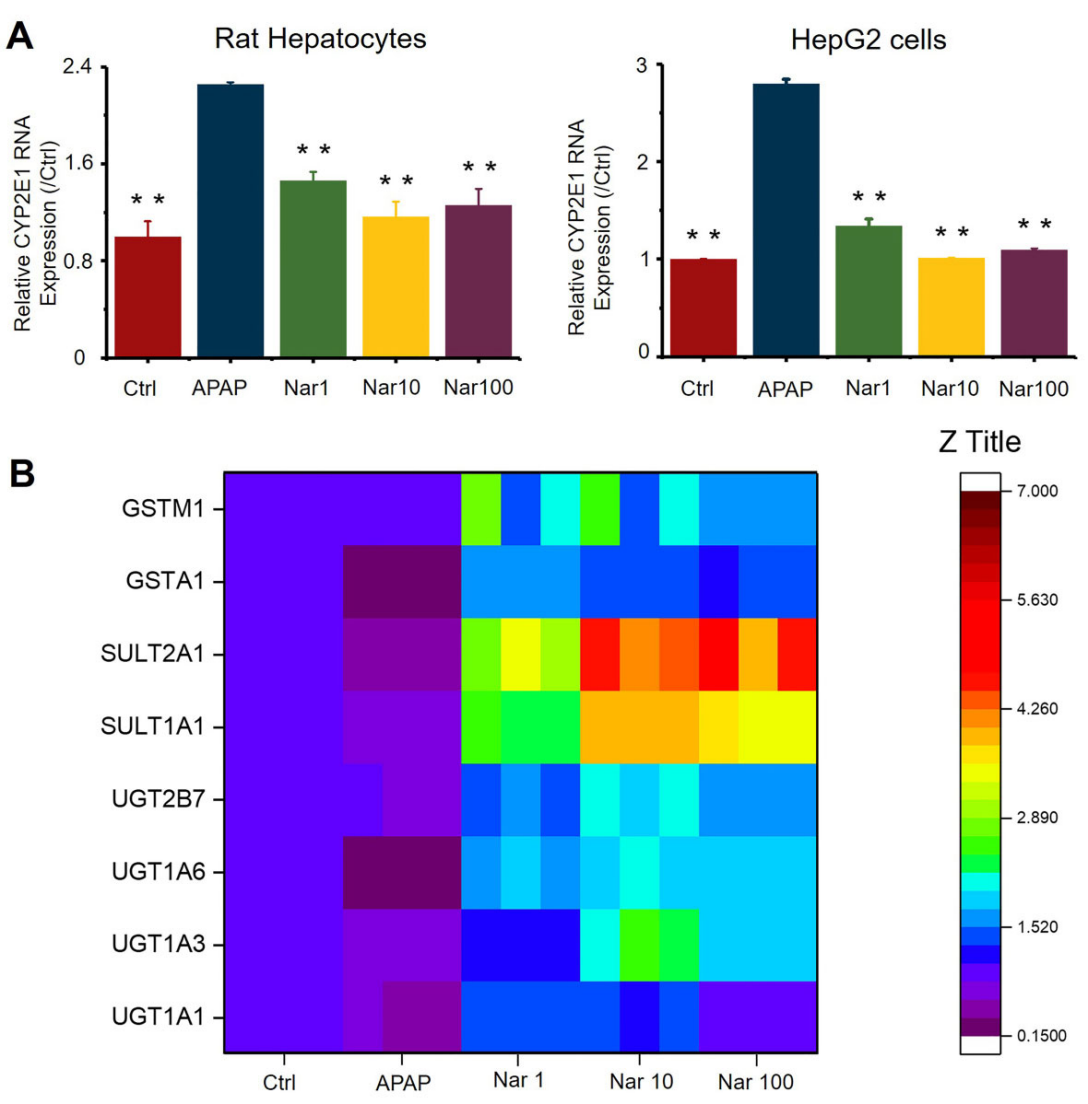
**A**, RT-qPCR results showed that Naringin (Nar) downregulated the gene expression of CYP2E1 in primary rat hepatocytes and HepG2 cells. **B**, Nar upregulated phase II enzymes involved in acetaminophen (APAP) metabolism in hepatocytes and in HepG2 cells. Data are reported as means±SD (n=3). **P<0.01 *vs* the APAP group (ANOVA).

According to the data of mRNA levels of CYP2E1 using RT-qPCR, we observed that APAP alone increased the expression of CYP2E1 in both types of cells. In addition, with dose-dependent Nar, CYP2E1 was significantly suppressed (Figure 3A).

### Nar increased the mRNA levels of phase II enzymes in HepG2 cells

Due to the significant differences in the levels of phase II enzymes between rat hepatocytes and human hepatocytes (23), we evaluated the changes in the levels of phase II enzymes caused by Nar in HepG2 cells. As indicated in Figure 3B, phase II enzyme levels were affected by even the lowest concentration of Nar. Compared to treatment with APAP alone, pretreatment with the highest dose of Nar resulted in increases of 46% (UGT1A1), 109% (UGT1A3), 146% (UGT1A6), 285% (SULT1A1), 407% (SULT2A1), 108% (GSTα1), and 76% (GSTm1) in the levels of these enzymes. Collectively, these results indicated that Nar induced upregulation of phase II enzymes mRNA expression levels in the setting of APAP-induced damage.

### Nar prevented and reversed APAP-induced mitochondrial and hepatocyte damage by regulating mitochondrial dynamics

**Figure 4:**
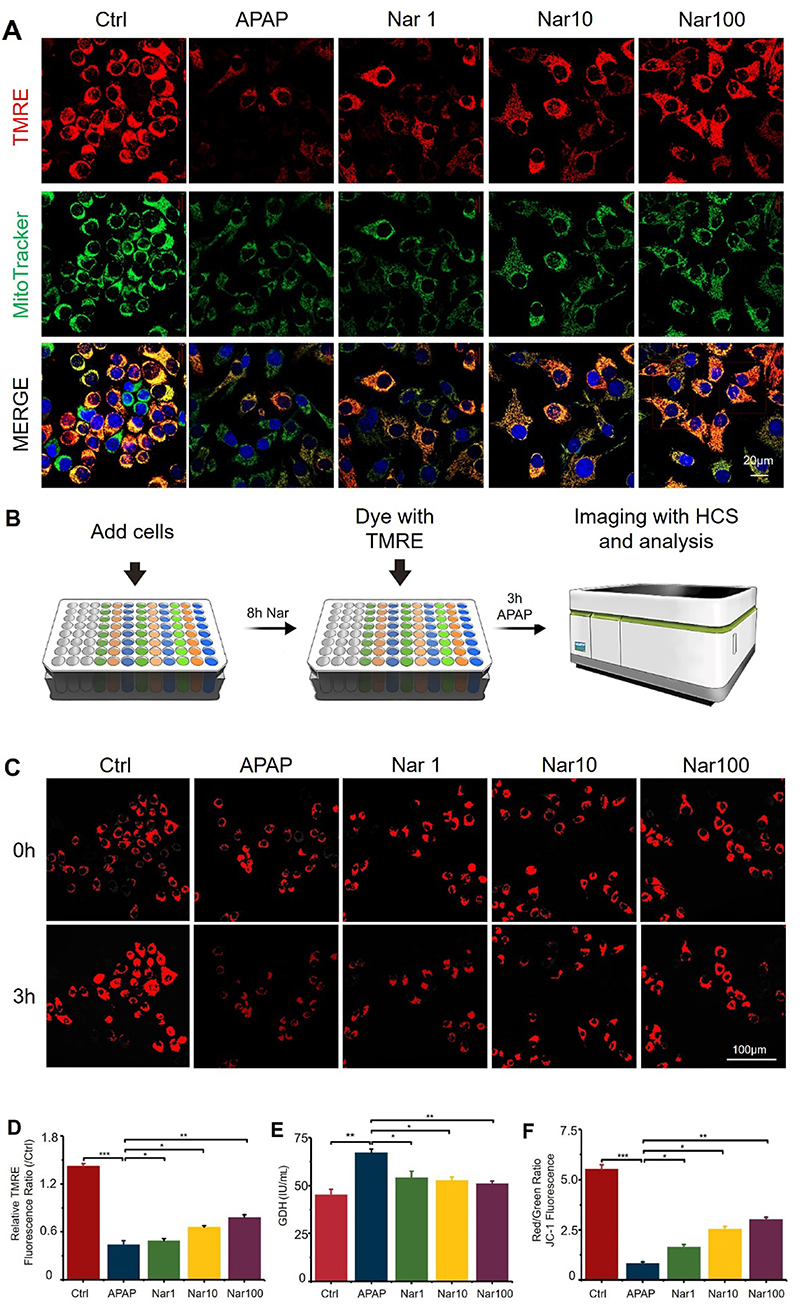
Naringin (Nar) prevented acetaminophen (APAP)-induced mitochondrial injury in HepG2 cells. **A**, Detection of changes in the TMRE/MitoTracker Green fluorescence signal by confocal microscopy (scale bar 20 μm). **B**, Flow chart of high-content analysis monitoring of MMP changes. **C** and **D**, TMRE intensity indicating the MMP at 0 and 3 h in different groups (scale bar 100 μm). **E**, Changes in the glutamate dehydrogenase (GDH) level, an index of mitochondrial injury, in different groups after 24 h APAP-induced injury. **F**, Red/green fluorescence ratio in JC-1-stained cells. Data are reported as means±SD (n=3). *P<0.05, **P<0.01, and ***P<0.001 *vs* the APAP group (ANOVA).

A decrease in the MMP is an important manifestation of mitochondrial injury and inhibiting the decrease in the MMP can prevent cell death. Previous studies have suggested that APAP reduces the MMP. To explore the effects of APAP on mitochondrial function, the MMP was measured by two approaches. Regardless of whether high-content analysis was used to monitor changes in the MMP (Figure 4B, C, and D) or confocal microscopy was used to detect changes in the TMRE/MitoTracker Green fluorescence signal (Figure 4A), APAP significantly reduced the MMP, while Nar increased the MMP in a dose-dependent manner.

To evaluate the effects of APAP and Nar treatment on mitochondrial depolarization in HepG2 cells, we used JC-1 assay, as shown in Figure 4F. The ratio of red to green fluorescence can be used to monitor the integrity of the mitochondrial membrane. After 8 h of treatment with 20 mM APAP, the red/green fluorescence ratio in HepG2 cells was significantly reduced, while Nar treatment increased the red/green fluorescence ratio in a dose-dependent manner.

**Figure 5:**
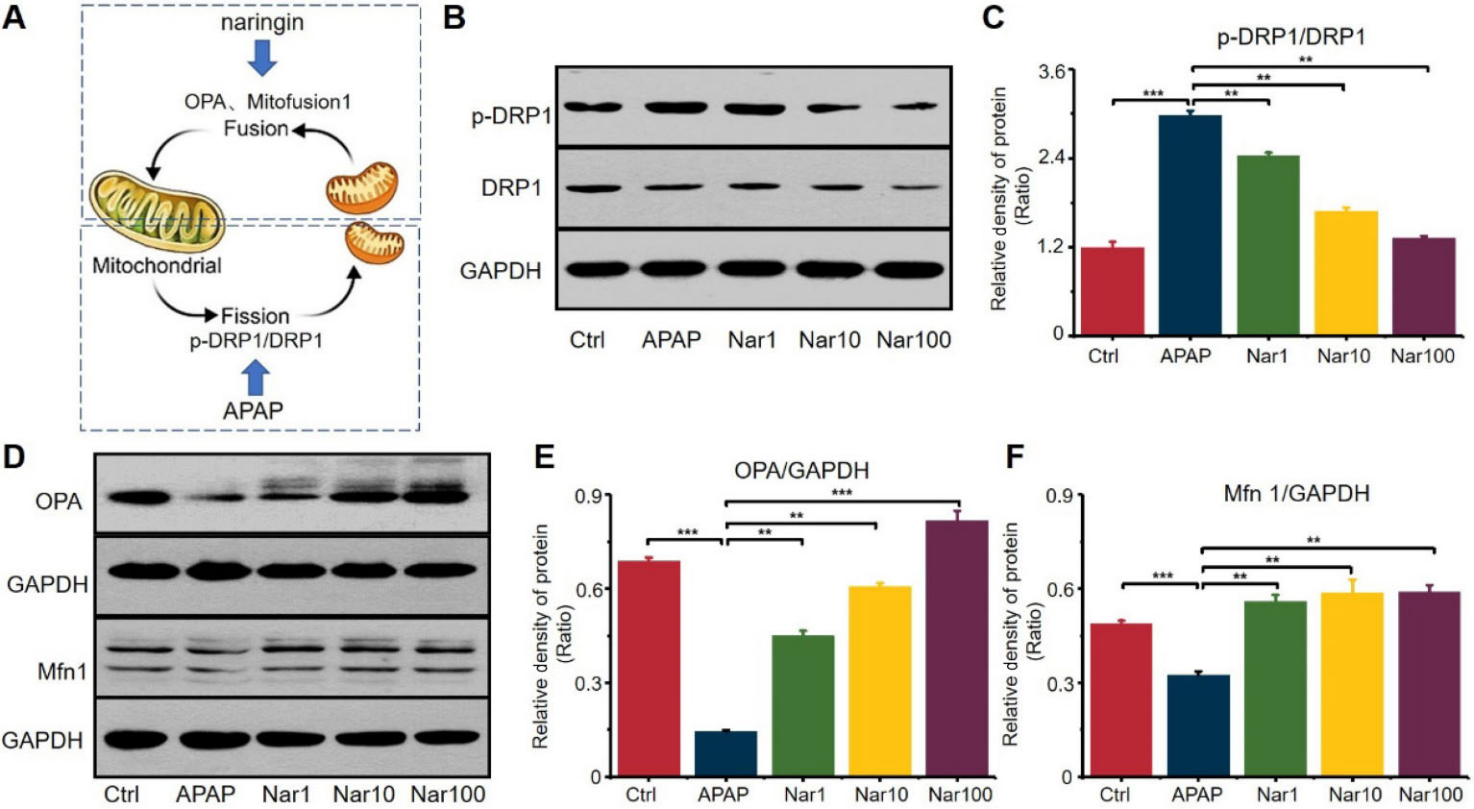
The effect of acetaminophen (APAP) and naringin (Nar) on mitochondrial dynamics. **A**, The equilibrium of mitochondrial dynamics. APAP promoted mitochondrial fission and Nar promoted mitochondrial fusion. **B** and **C**, Western blot analysis of the effects of Nar and APAP on mitochondrial fission (p-DRP1/DRP1). **D**, **E**, and **F**, Western blot analysis of the effect of Nar and APAP on mitochondrial fusion (OPA, Mfn 1). Data are reported as means±SD (n=3). **P<0.01 and ***P<0.001 *vs* the APAP group (ANOVA).

To investigate the protective effect of Nar against APAP-induced mitochondrial malfunction, mitochondrial dynamics were examined in HepG2 cells. Western blot analysis showed that APAP significantly decreased the expression levels of the fusion proteins Mfn1 and Opa1 to 33.7 and 78.9% of the levels in control cells (Figure 5D, E, and F). These results demonstrated that APAP downregulated the fusion machinery. Despite the presence of APAP, the administration of Nar markedly increased Mfn1 expression by 66% compared with that in control cells (Figure 5D, E, and F). Nar also prevented the APAP-induced decrease in Opa1 expression, increasing its expression by 69% compared with that in control cells (Figure 5D and F). These results showed that Nar inhibited the APAP-induced decrease in mitochondrial fusion protein expression. In addition, APAP affected the activity of the fission protein Drp1, as measured by the ratio of p-Drp1 (Ser616) to total Drp1 (Figure 5B and C). Taken together, these results showed that APAP induced mitochondrial fragmentation via downregulation of mitochondrial fusion proteins. Nar promoted mitochondrial fusion, thus ameliorating mitochondrial malfunction under APAP-induced oxidative stress.

### Nar treatment induced phosphorylation of AMPK and upregulation of Nrf2 in APAP-induced hepatotoxicity

**Figure 6:**
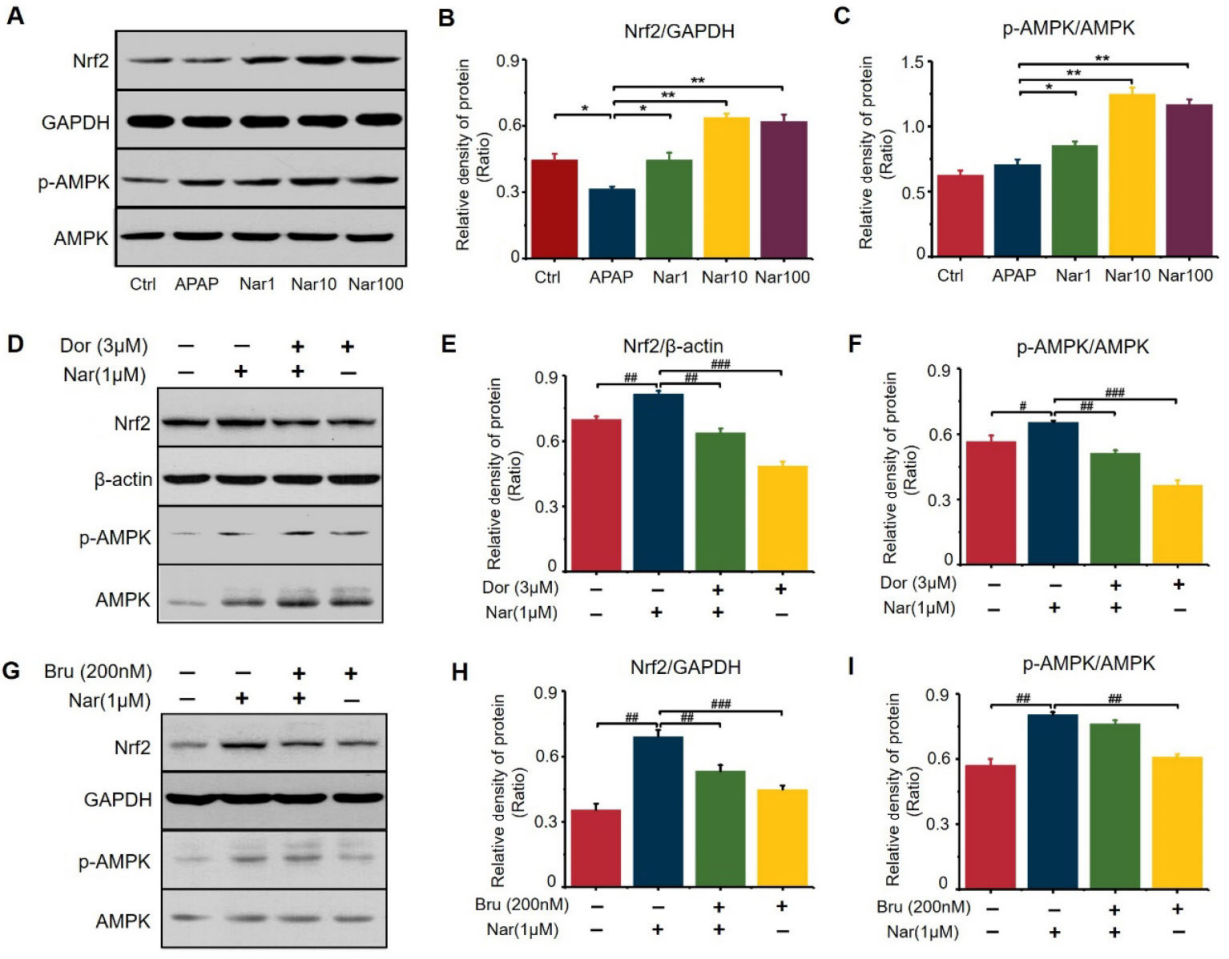
Naringin (Nar) activated AMPK/Nrf2 signaling in HepG2 cells. **A**, **B**, and **C**, Western-blot results of Nrf2 and pAMPK/AMPK expression. **D**, **E**, and **F**, HepG2 cells were treated with dorsomorphin (Dor) (3 μM) and Nar (1 μM) for 8 h, and proteins (Nrf2 and pAMPK/AMPK) were detected by western blotting. **G**, **H**, and **I**, HepG2 cells were treated with brusatol (Bru) (200 nM) and Nar (3 μM) for 8 h, and proteins were detected by western blotting. Data are reported as means±SD (n=3). *P<0.05, **P<0.01 *vs* the APAP group. ^#^P<0.05, ^##^P<0.01, and ^###^P<0.001 compared with cells treated only with Nar (ANOVA).

Because Nrf2 plays a key role in regulating the expression of numerous antioxidant enzymes (21), we sought to determine whether Nar treatment can upregulate Nrf2 (Figure 6A and B). Our experiments indicated that Nar efficiently upregulated the expression of Nrf2. Compared to those in the APAP group, the protein levels of Nrf2 were increased by 101 and 105% in the groups pretreated with 10 and 100 μM Nar, respectively. Nar promoted the phosphorylation of AMPK in HepG2 cells (Figure 6A and C). To further determine the link between Nar-mediated Nrf2 and AMPK, HepG2 cells were incubated with dorsomorphin (an inhibitor of AMPK) or brusatol (an inhibitor of Nrf2). Then, we verified the effects of these two inhibitors on Nrf2 expression and AMPK phosphorylation and found that dorsomorphin effectively blocked Nar-mediated Nrf2 activation and AMPK phosphorylation (Figure 6D, E, and F). However, brusatol had no significant effect on Nar-mediated AMPK phosphorylation, demonstrating that AMPK can act as an upstream regulator of Nrf2 (Figure 6G, H, and I).

## Discussion

APAP-induced liver injury is the main reason of acute liver failure in developed countries, and existing treatments are limited. Therefore, there is an urgent need to develop new therapies (1). Normally, oxidation helps balance signal transduction, but excessive oxidant expression leads to disease process. Extreme oxidative stress is crucial in APAP-induced liver injury (6). Nar has shown the ability to reduce oxidative stress in previous research (19,20). APAP can be metabolized by the CYP2E1 enzyme to NAPQI, and chemically and enzymatically combined with GSH, which may lead to lipid peroxidation, nitrated protein, and subsequent liver cell damage. Studies have shown that excessive APAP can reduce the activity of antioxidant enzymes, and some drugs have been proven to restore part of the enzyme's function (13,14,24). In this study, APAP reduced the concentrations of GSH and SOD in hepatocytes, and increased the concentrations of MDA and ROS, all of which are indicators of oxidative stress. Antioxidant enzyme levels increased in cell groups pretreated with Nar, while MDA concentration decreased. The data showed that an increase in the level of antioxidant enzymes was the result of the protective effect of Nar against APAP-induced liver cell damage. APAP increased the expression of CYP2E1 in the liver. Nar significantly inhibited the expression of CYP2E1 and increased the levels of antioxidant enzymes. This may be due to the strong antioxidant effect of Nar. The catalytic metabolism of phase II enzymes such as UGTs, SULTs, and GST plays an important role in the nontoxic metabolism of APAP. APAP was detoxified by the phase II enzymes in the liver. Approximately 85% of APAP is transformed into APAP-sulfur and APAP-gluc by phase II enzymes including UGTs and SULTs (13). In this study, there were 7 kinds of phase II enzymes involved in the detoxification of APAP, which were all main enzymes and main subtypes of UGTs, SULTs, and GSTs. This study confirmed that mRNA expression of most of phase II enzymes increased in a dose-dependent manner with Nar. At the same time, Nar significantly increased the mRNA levels of UGT1A9 and SULT2A1. These results indicated that regulation of phase II enzymes might be involved in the mechanisms by which Nar prevents liver cell damage.

Nrf2 activation is the key to inducing phase II enzyme expression. The importance of Nrf2 is very clear. There are reports showing that in Nrf2-deficient mice, the level of phase II enzyme genes is significantly reduced (25,26). The experimental results showed that Nar significantly upregulated the expression levels of SULTs and UGTs, as shown in Figure 3. In addition, there are reports showing that Nrf2-deficient mice are more susceptible to APAP-induced liver injury, which indicates that Nrf2 is a target for liver protection (25). Nrf2 is a CNC-bZIP protein that is considered to be the main regulator of oxidative defense (27). Pretreatment with Nar induced the upregulation of Nrf2 protein level. We also tested how AMPK/Nrf2 signaling pathway affected APAP-related phase II enzymes. We used RT-qPCR to verify that Nrf2, instead of AMPK, upregulated the expression of related phase II enzymes (Supplementary Figure S1). Some reports have found that Nrf2 modulates the synthesis of GSH (24). Nar increased the GSH levels by upregulating Nrf2 expression. These results indicated that Nrf2 participates in the detoxification and antioxidant signaling pathways induced by Nar.

Mitochondrial malfunction plays an important role in APAP-induced hepatotoxicity (5). In APAP-induced liver injury, AMPK activation is beneficial for hepatocyte survival and therapeutic outcome to minimize mitochondrial damage and/or enhance mitochondrial function (28). The mitochondrial quality control system, which regulates mitochondrial fusion/fission dynamics, is helpful for keeping mitochondria healthy within cells (29). The outer membrane fusion protein Mfn1 and the inner membrane fusion protein Opa1 are involved in mitochondrial fusion, while the fission protein Drp1, a protein that only has biological activity after phosphorylation, participates in mitochondrial fission. When a mitochondrion is slightly damaged, it will fuse with a healthy mitochondrion to replenish the damaged components and maintain the normal function. When the mitochondria are severely damaged and overloaded, they will actively fission, thereby producing a healthy and a damaged mitochondria and facilitating their separation. Healthy mitochondria can refuse and perform their functions again. In addition, the severely damaged mitochondria are degraded to produce biomaterials that can be further used to synthesize new mitochondria. Through these mechanisms of “quality control”, mitochondria diminish or eradicate injury from intra- and extracellular stress. APAP can promote the expression of fission proteins (phosDrp/Drp), impair mitochondrial fusion, lead to mitochondrial fragmentation and malfunction, and ultimately result in hepatocyte damage (28). Some studies have reported that the activation of AMPK maintains normal mitochondrial morphology and function and prevents drug-induced mitochondrial and hepatocellular injury (4,30). Nar can activate AMPK and reverse the early stage of APAP-induced mitochondrial and hepatocellular injury through several mechanisms including the promotion of mitochondrial fusion by increasing expression of fusion proteins (i.e., Mfn1 and Opa1), and the downregulation of the expression of fission proteins (phosDrp/Drp). These mechanisms partly restore MMP and maintain mitochondrial function. These observations suggest that Nar activates AMPK to help reverse drug-induced mitochondrial injury and constitutes a viable approach for treating APAP-induced liver injury.

Recent studies have shown that AMPK is involved in the activation of Nrf2 mediated by different compounds (30,31). To verify whether AMPK mediates Nrf2 activation by Nar, we measured the effect of Nar on AMPK phosphorylation in HepG2 cells. Nar could upregulate the expression of pAMPK in a dose-dependent manner, demonstrating that AMPK may activate Nrf2 signaling pathway. In this study, the plant product Nar notably resisted APAP-induced decrease of cell viability in primary rat hepatocytes and HepG2 cells. The hepatoprotective effect of Nar against APAP injury was associated with inhibition of ROS production. Nar activates pAMPK, leading to the increase of the vital anti-oxidant regulator, Nrf-2.

**Figure 7:**
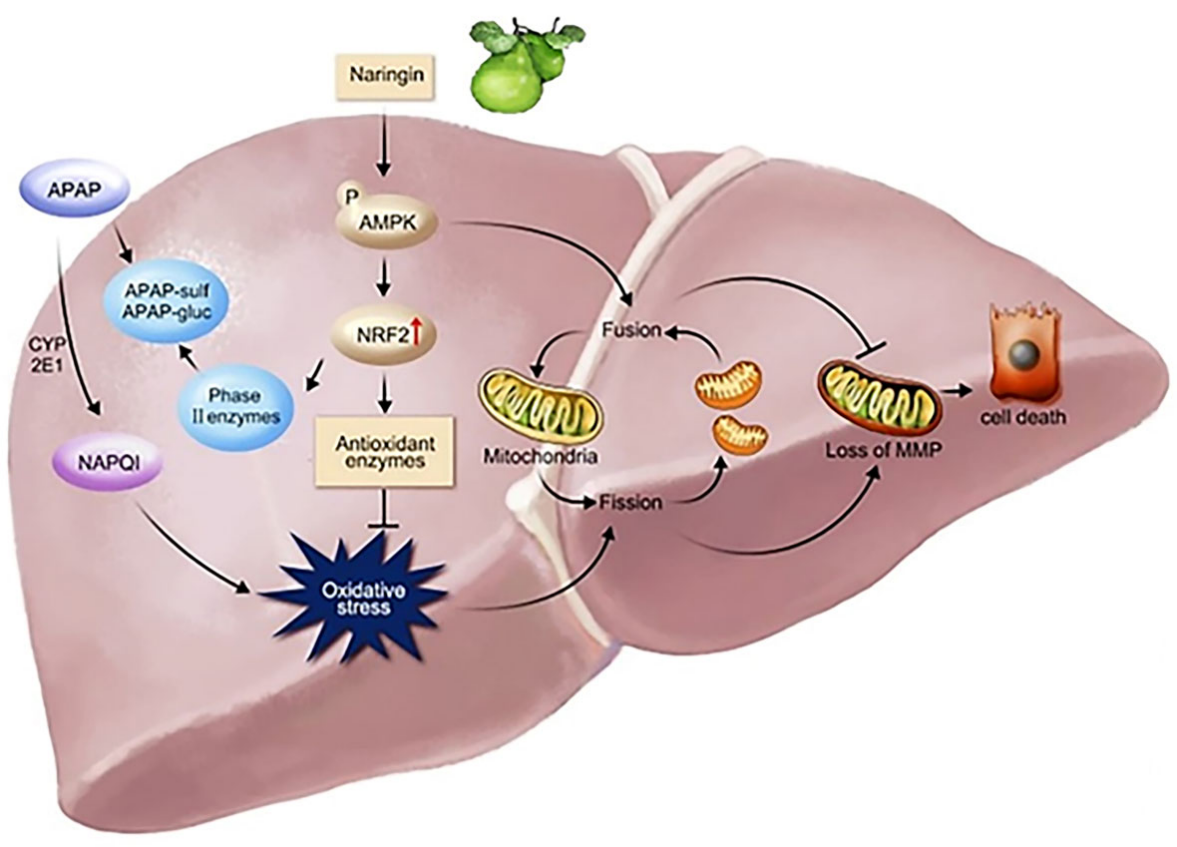
Mechanisms by which naringin (Nar) can prevent acetaminophen (APAP)-induced hepatocyte damage.

The results of this study suggested that Nar can prevent APAP-induced hepatocyte damage in multiple ways, as shown in Figure 7. The mechanism of Nar-mediated expression of toxic pathways is the activation of Nrf2 to upregulate the expression of antioxidant enzymes and phase II enzymes, thereby accelerating the non-toxic metabolism of APAP. Moreover, Nar can restore MMP and maintain mitochondrial function by activating AMPK and improving mitochondrial fusion (Mfn1/Opa1).

In summary, our experiments suggested that Nar prevented APAP-induced hepatotoxicity through the inhibition of oxidative stress, the upregulation of antioxidant enzymes and phase II enzymes, and the promotion of mitochondrial fusion. The compound might be a potential protective agent against APAP-induced liver injury. To improve clinical significance of our findings, further studies need to investigate the therapeutic effect of Nar against the disease *in vivo*.

## Supplementary Material

Click here to view [pdf].
